# Draft Genome Sequences of Micrococcus luteus MFP06 and MFP07, Isolated from the Skin of Healthy Volunteers

**DOI:** 10.1128/MRA.00545-20

**Published:** 2020-06-18

**Authors:** Djouhar Souak, Amine M. Boukerb, Magalie Barreau, Cecile Duclairoir-Poc, Marc G. J. Feuilloley

**Affiliations:** a Laboratoire de Microbiologie, Signaux et Microenvironnement (LMSM) EA412, Université de Rouen Normandie, Evreux, France; Indiana University, Bloomington

## Abstract

We report the draft genome sequences of two Micrococcus luteus strains, MFP06 and MFP07, isolated from human skin. The genome assemblies consist of 2,480 and 2,417 kbp with 2,337 and 2,240 coding sequences, respectively. The genomes contain genes potentially involved in osmotic stress tolerance, DNA repair, monoacylglycerol hydrolysis, and beta-lactone synthesis.

## ANNOUNCEMENT

Micrococcus luteus is a high-GC-content, Gram-positive, strictly aerobic coccus typically occurring in tetrads and phylogenetically affiliated with the family *Micrococcaceae* in the phylum *Actinobacteria*. This bacterium is found in environments such as soil (1), air (2), and human skin (3, 4). M. luteus is also known as an opportunistic pathogen involved in severe infections such as meningitis and septic shock in immunocompromised patients with reported antibioresistance (5). Despite this opportunistic behavior, little is known about its role within the skin microbiome.

M. luteus strains MFP06 and MFP07 were collected under the control of the Bio-EC CRO (Longjumeau, France) and according to the French and European ethical directives (ARS Biomedical Research Agreement 2012-12-010, Bioethics Agreement DC-2008-542) (6). These strains were isolated by swabbing the right antecubital fossa of an adult woman (50 to 65 years old) and the right scapula of an adult man (50 to 65 years old), respectively. Bacterial colonies cultured on tryptic soy agar (TSA) at 37°C were identified as M. luteus by analysis of their total proteome using an Autoflex III matrix-assisted laser desorption ionization–time of flight (MALDI-TOF) mass spectrometer coupled to the MALDI-Biotyper 3.0 algorithmic system (Bruker, Marcy-l’Étoile, France) (6). For genome sequencing, DNA was extracted from an overnight culture in tryptic soy broth (TSB) at 37°C with a genomic extraction kit (GeneJET genomic DNA purification kit, catalog number K0721; Thermo Scientific) following the supplied procedure, with pretreatment using a lysis solution (20 mM Tris-HCl [pH 8.0], 2 mM EDTA, 1.2% Triton X-100, and 20 mg/ml lysozyme). Library preparation and sequencing were conducted at the LMSM genomics platform (LMSM Evreux, University of Rouen Normandy). Briefly, libraries were prepared with the Nextera XT DNA sample preparation kit (Illumina, USA) and sequenced on an Illumina MiSeq system (MiSeq reagent kit v.3, 600 cycles), generating 1,604,848 and 2,745,926 high-quality raw paired-end (PE) 250-bp reads, respectively.

All bioinformatic tools were used with default parameters unless otherwise stated. Reads were quality screened and trimmed with FastQC v.0.11.8 (7) and Trim Galore v.0.6.2 (8), respectively. Genome assembly was conducted using Unicycler v.0.4.7 (9), and sequences were assessed for contamination with CheckM v.1.1.2 (10). Automated gene predictions and functional annotations were performed using the NCBI Prokaryotic Genome Annotation Pipeline (PGAP) (11). Coding genes for proteins possibly involved in secondary metabolite production were identified with antiSMASH v.5.1.2 (12). The draft genomes of MFP06 and MFP07 consisted of 2,480,672 and 2,417,497 bp in 221 and 165 contigs (*N*_50_, 19,847 and 30,452) with 147.6 and 219.4× mean coverage, respectively. The GC content for both draft genomes was 72.96%. There are 2,337 and 2,240 protein-coding genes, 51 and 52 tRNA genes, 1 copy each of 5S, 16S, and 23S rRNA genes, and 36 and 34 insertion sequence (IS) elements, respectively. Genes coding for monoacylglycerol lipases involved in lipid metabolism (13), l-ectoine synthase, which helps resist shifts in salt concentration (14), and a biosynthetic gene cluster (BGC) coding for a beta-lactone (15) were also detected. Future detailed analysis of these loci and the genomic characterization of these strains will provide further information about adaptation and success of *M. luteus* on human skin.

### Data availability.

Sample information, genomic assembly and annotation, and raw sequences are accessible under the NCBI BioProject number PRJNA626598. The whole-genome shotgun (WGS) projects are available under the GenBank accession numbers JABBWS000000000 and JABBWT000000000, and the SRA accession numbers are SRR11574543 and SRR11574542.
